# Cognitive dissonance as a reason for low perceived HIV risk among Black women

**DOI:** 10.3389/fsoc.2024.1498383

**Published:** 2025-01-10

**Authors:** Mandy J. Hill

**Affiliations:** University of Texas Health Science Center at Houston, Houston, TX, United States

**Keywords:** sexual health, cognitive dissonance, Black women, culture, perceived risk and normative influences, coping strategies, human immunodeficiency virus

## Abstract

Existing HIV-related literature affirms that Black women in the US have a low perceived risk of HIV. Yet, Black women consistently experience higher HIV incidence than other women. The ability of HIV risk perception to influence HIV prevention behaviors remains unclear. Lack of knowledge is often described as the primary driver of a low perceived risk of contracting HIV. What if the primary driver is not lack of knowledge? Instead, it is possible and even likely, that cognitive dissonance became a commonly used coping strategy for survival among Black women whose social standing hinges, in part, on the independent ability to maintain romantic partnerships while doubling in purpose as a primary driver for low perceived risk of HIV. The three key points of this commentary are that underpinnings of low perceived risk of HIV among Black women exist, cognitive dissonance is a likely byproduct of reconciling cultural norms with self-identity, and there is a permanence in disconnect between actual and perceived risk of HIV among Black women. To achieve sexual health equity, researchers must enhance awareness into the nuanced reasons that low perceived risk of HIV persist.

## Introduction

Over the last 35 years, low perceived risk of HIV has been discussed as a prevalent qualitative finding among Black women across the sexual health literature (Younge et al., 2010; Amaro, and Love, sex, and power, 1995; Chapin, 2001; Chapin, 2000; Ellen et al., 2002; Ellen et al., 1996; Ferguson et al., 2006; Ford et al., 2006; Kalichman and Cain, 2005; Mays and Cochran, 1988; Nydegger et al., 2020; Budge et al., 2023). This finding has been linked to condomless sex (Calabrese et al., 2019; Gilbert et al., 2021; Hill et al., 2022; Hill et al., 2023; Patel et al., 2019; Pratt et al., 2022), multiple sexual partners (Calabrese et al., 2019; Adimora et al., 2002; Gerend and Magloire, 2008; Grimley and Hook, 2009; Ho et al., 1998; Khan et al., 2009; Nydegger et al., 2020; Sewell and Blankenship, 2019; Sly et al., 1997; Tillerson, 2008; Wagstaff et al., 1995), and most recently low PrEP initiation rates (Hill et al., 2022; Cheek et al., 2022; Flash et al., 2018; Nydegger et al., 2020; Pyra et al., 2022; Willie et al., 2021). Literary findings illustrate that having low perceptions of HIV vulnerability is often identified among Black women who report personal sexual behaviors that place most individuals at significant risk for HIV. Researchers and interventionists have yet to discover fundamental drivers for reports of low perceptions of risk. In fact, the literature has largely attributed this common factor to a lack of knowledge (Kiviniemi et al., 2018). This commentary aims to challenge the notion that a lack of knowledge is the sole reason for the prevalence of a low perceived risk of HIV vulnerability among diverse groups of Black women in the US. The literature has failed to distinguish differences in perceptions of HIV risk by demographic profiles of Black women by education, income, insurance coverage, social support systems, and/or family dynamics. The proposal here is that an undiscovered aspect of the low perceived HIV risk among Black women in the US relates to cultural factors that may extend beyond demographic considerations.

### Investigating the underpinnings of low perceived risk of HIV among Black women

Black women have been indoctrinated in a culture that embodies different practices, values, and behaviors. Some of those complex factors esteem partnership as an extension of personal value (Henry, 2013). Henry (2013) described the emphasis of researchers who have studied identity development among women, leading to her concluding remarks where ‘it could be surmised that the dating decisions of Black women are influenced in part by their experiences at particular stages of racial and gender identity formation’ (Henry, 2008). For Black women in the US, race and gender identity relegates this segment of the population as least valuable on the societal hierarchical scale of human life. (Rodriguez, 2018). Vulnerability to health risks, which include HIV vulnerability, are a consequence of this social stratification, whereby being relegated as less valuable in society hinders access to education, employment, healthcare, while driving stigma, poverty, cultural norms, and social beliefs (Thapa et al., 2021). Of the nine studies assessed through the systematic review referenced in the prior sentence, one of those study’s ‘asserts that disadvantaged women have a higher chance of severe illness and without any improvement in caste and class barriers, improved health resources and outcomes are almost impossible.’ Rosenthal and Lobel (2016) said that ‘the assumptions people make about Black women have a wide range of implications for the ways that Black women are treated in our society’.

The lingering by product of societal norms whereby a racial and gender identified group is deemed to be of low human value is countered by familial norms where those same group members are esteemed highly within family units with purposeful intention to insulate and preserve the value of each individual member. This approach offers each individual member a level of protection, simply by being a part of the whole. It also afforded Black people freedom, self-determination, autonomy, and agency (Smith et al., 2019; Waterman, 1981). Findings of empirical studies of cultural values with African American and Black adolescent girls ‘found that collectivistic values operationalized as collective work and responsibility, cooperative economics, and self-determination, were associated with higher levels of self esteem, perceived social support, and life satisfaction’ (Henry, 2013). These viewpoints have been passed down from one generation to the next with African American and Black people here in the United States (Smith et al., 2019; Constantine et al., 2006).

When women become of the age in society to become partnered, there is a communal expectation reinforced by the matriarchs, including mothers, grandmothers, aunts, family friends, church members, etc. to motivate the ‘coming of age’ woman to prioritize being selected as a partner (Salisu and Dacus, 2021). Salisu and Dacus (2021) state that ‘Black women have strived to survive in a society that created and has perpetuated an unequal system of social and economic deprivation of their Black husbands, in heterosexual relationships.’ The qualities of the partner are general and have traditionally hyper-focused on the partner’s ability to provide support and stability. Qualities such as fidelity and trust are rarely mentioned. Cultural conditioning of partner selection in some cases is reinforced by the teachings of women in the female lineage of Black women who are indoctrinated to ‘accept the role of the strong Black woman’ and ‘embrace this role and frequently make sacrifices because their life choices were affected by those of their families’ (Smith et al., 2019). When indoctrinated within this culture, Black women are often met with the need to identify strategies to cope with the pressure while adjusting to a new found independence in determining one’s life course (Salisu and Dacus, 2021). This is where cognitive dissonance has the optimal opportunity to make an appearance and establish a permanent foundation.

### Cognitive dissonance as the unintended byproduct of reconciling cultural norms with self-identity

Cognitive dissonance is the ‘mental discomfort that results from holding two conflicting beliefs, values, or attitudes’, especially as relating to behavioral decisions and attitude change (Cherry, 2022). Feelings associated with cognitive dissonance can morph into feelings of low self-esteem and low self-worth. Cognitive dissonance can be a powerful internal influence on behaviors and actions of an individual. Conversely, Black matriarchs can be a powerful external influence on behaviors and actions of Black people in the US, especially Black women who are coming of age. Through the power of influence through relational interactions, Black matriarchs often build social network power that solidify their centralized roles in families and their communities (Salisu and Dacus, 2021).

When cognitive dissonance arrives, Black women may have to grapple with the feelings of low self-esteem while placating the demands of the matriarchs to be partnered. Salisu and Dacus (2021) described the experience of some Black women who live in a paradox where they struggle with trying ‘to balance the expectations of their roles they occupied in society with their own identity as a sexual being’. When prioritizing the needs of others, which in this case are elders and respected women in the community (Rosenthal and Lobel, 2016), a coping strategy to please themselves may result in adopting a low perceived risk of HIV in order to garner the coveted personal value bestowed upon an individual who is a part of a complex culture in society that in part, values when a Black woman is ‘chosen’ by a male partner. The stigma of not being ‘chosen’ in society can be accompanied by a negative stereotype (e.g., a harmful belief or disparaging opinion about a group of people that falsely associates negative characteristics to them), which ‘have important consequences in many realms of life’; ‘stereotypes have a connection to decision-making’ (Constantine et al., 2006). The mental exercise of showcasing oneself as ‘desirable’ can become a survival strategy in an effort to stave off judgment and ridicule while ascertaining and preserving social standing in a multifaceted culture with components that values partnership, with no metric of quality assurance.

Sewell and Blankenship (2019) identified a complex interplay between risk perception, risk behaviors, and stigma related to HIV among a female cohort comprised of individuals who were mostly Black (93.0%) and impoverished (87.7%). For the past 20 years, the degree of influence that HIV risk perception has had on decisions to change patterns of engagement in behaviors that are linked to reasons for contracting HIV remains unclear and inconsistent (Sewell and Blankenship, 2019; Prata et al., 2006). Scientists aim to seek clarity in the known associations. Women poverty (*p* = 0.010), employment (*p* = 0.012), insurance (*p* = 0.024) and homelessness status (*p* < 0.001) were all significantly associated with their level of HIV risk perception (low, medium, or high) (Sewell and Blankenship, 2019). Most of the participants lived in poverty, were unemployed, and had health insurance. Specifically, women who experienced homelessness in the last 12 months were most likely to have a high perceived risk of HIV versus low risk (68.1% vs. 35.8%, *p* < 0.001). Knowledge of a male sex partner’s HIV status at last intercourse was low among women with low HIV risk perception (68.7%, *p* = 0.007) and most of them reported condomless anal or vaginal sex within the last year (86.7%, *p* = 0.025). HIV stigma and discrimination did not significantly influence HIV risk perception among a sample of mostly Black women in Detroit, MI within the United States (Sewell and Blankenship, 2019). Sewell and Blankenship (2019) described how Black women who engaged in sexual behaviors that included reasons for HIV vulnerability were more likely to experience stigma. As a result, those women were likely to perceive those experiences as ‘negative’ in association with HIV risk. Some researchers hypothesize that when women have negative experiences in relationships, these experiences are accompanied by an understanding and acceptance of HIV vulnerability, which may influence Black women to disassociate themselves with HIV risk (Sewell and Blankenship, 2019). Sewell and Blankenship (2019) found that ‘many women with low HIV risk perception still engaged in high-risk behaviors’ (Sewell and Blankenship, 2019). By linking the HIV risk to the negative experience, the women unlink themselves from the HIV risk when they unlink themselves from the negative experience or from the partner who they perceive as causing the negative experience. Pharr et al. (2015) found that ‘HIV risk perception may not ultimately lead to reductions in high-risk behaviors, as socioeconomic, sociocultural and structural constraints represent an external locus of control that high-risk populations face, but cannot necessarily overcome’ (Pharr et al., 2015). The translation of using this temporary fleeting strategy into a permanent dissociation of risk removes the responsibility and accountability to the woman’s personal sexual health. This likely leads to a permanence of low perceived risk of HIV. This allows for a permanence in vulnerability to HIV and subsequently, an abandonment of self-protection.

### The permanence in disconnect between actual and perceived risk of HIV among Black women

Human beings tend to shy away from or avoid feelings of discomfort (Connelly, 2009). One of the ways in which we minimize discomfort is to manifest and make our ideals, fantasies, and figments of our imaginations seemingly real. This helps us to avoid facing inconsistencies between beliefs and behavior. Over time, when the avoidance becomes the default behavior, the distinction between knowing whether what was imagined versus what was real becomes unclear and what was once seemingly real becomes real. When what was once seemingly real becomes the default perception, it becomes misconstrued as what is actual. This misconstrued information between perception and actual risk has likely become a permanent disconnect between actual and perceived risk of HIV among Black women. The strength of this disconnect, or cognitive dissonance, can be influenced by factors that can include the importance one attaches to a belief and/or the quantity of dissonant beliefs (Cherry, 2022).

## Discussion

Consideration of such psychologically rooted complex models highlighting multifactorial influences from societal and social constructs that mediate or moderate risk perceptions among HIV-negative Black women in the US who engage in heterosexual sex are necessary to fully understand correlations that may appear illogical on the surface.

Precursors for women’s HIV risk perceptions must be identified in order to effectively bridge the gap between eligibility for HIV prevention services, particularly pre-exposure prophylaxis (PrEP), and access to and uptake of those services among Black women. Even among 112 Black women who are using PrEP, the decision to continue PrEP treatment was based on their perceived HIV risk, resulting in only 18% who were identified as persistent PrEP users (Pyra et al., 2022).

The lack of congruence between behaviors that are aligned with HIV transmission routes, HIV risk perception, and expectations of negative experiences linked to an HIV status suggests that more research is needed to better understand these pathways among Black women in the United States (Sewell and Blankenship, 2019). If cognitive dissonance is indeed one of the determinants of low perceived HIV risk among Black women, overcoming this communal dissociation is required if we are to realize ending the HIV epidemic. Advancing sexual health equity for all, especially for Black women in the US, will require us to look beneath the surface to seek and understand the reason for the decision that leads to the behavior (i.e., taking PrEP or not taking PrEP). Once we have sought and understood the reason, we must honor and respect it, then heal it before we set forth to rectify it and change it. Reversing the trend in the uptake of effective sexual health services among Black women will require consistency, commitment, and a continuous investment in actualizing sexual health equity among a group of women who have likely had to collectively dissociate to survive generations of marginal access to full humanity.

## Data Availability

The original contributions presented in the study are included in the article/supplementary material, further inquiries can be directed to the corresponding author/s.

## References

[ref1] Adimora A. A. Schoenbach V. J. Bonas D. M. Martinson F. E. Donaldson K. H. Stancil T. R. (2002). Concurrent sexual partnerships among women in the United States. Epidemiology 13, 320–327. doi: 10.1097/00001648-200205000-00013 11964934

[ref2] Amaro H. Love, sex, and power (1995). Considering women's realities in HIV prevention. Am. Psychol. 50, 437–447. doi: 10.1037/0003-066X.50.6.437 7598292

[ref3] Budge M. Opara I. Weser V. U. Sands B. E. Hieftje K. D. (2023). Black adolescent Females' perceptions of PrEP for HIV risk reduction. J. Int. Assoc. Provid. AIDS Care 22:23259582231206934. doi: 10.1177/23259582231206934, PMID: 37853731 PMC10588402

[ref4] Calabrese S. K. Willie T. C. Galvao R. W. Tekeste M. Dovidio J. F. Safon C. B.. (2019). Current US guidelines for prescribing HIV pre-exposure prophylaxis (PrEP) disqualify many women who are at risk and motivated to use PrEP. J. Acquir. Immune Defic. Syndr. 81, 395–405. doi: 10.1097/QAI.0000000000002042, PMID: 30973543 PMC6594908

[ref5] Chapin J. R. (2000). Third-person perception and optimistic Bias among urban minority at-risk youth. Commun. Res. 27, 51–81. doi: 10.1177/009365000027001003

[ref6] Chapin J. (2001). It Won't happen to me: the role of optimistic Bias in African American Teens' risky sexual practices. Howard J. Commun. 12, 49–59. doi: 10.1080/10646170119661

[ref7] Cheek J. B. Feldman M. B. Montero N. Gambone G. F. Hoffman S. Blackstock O. J. (2022). Pre-exposure prophylaxis (PrEP) initiation among Black and Latina cisgender women receiving HIV prevention care coordination Services in new York City. AIDS Behav. 26, 3174–3184. doi: 10.1007/s10461-022-03661-1, PMID: 35362904

[ref8] Cherry K. (2022). Cognitive dissonance and the discomfort of holding conflicting beliefs: how we resolve our internal conflicts. Dotdash Meredith Publishing. Available at: https://www.verywellmind.com/what-is-cognitive-dissonance-2795012 (Accessed January 3, 2025).

[ref9] Connelly J. E. (2009). The avoidance of human suffering. Perspect. Biol. Med. 52, 381–391. doi: 10.1353/pbm.0.0095 19684373

[ref10] Constantine M. Alleyne V. Wallace B. Franklin-Jackson D. (2006). Afrocentric cultural values: their relation to positive mental health in African-American adolescent girls. J. Black Psychol. 32, 141–154. doi: 10.1177/0095798406286801

[ref11] Ellen J. M. Adler N. Gurvey J. E. Millstein S. G. Tschann J. (2002). Adolescent condom use and perceptions of risk for sexually transmitted diseases: a prospective study. Sex. Transm. Dis. 29, 756–762. doi: 10.1097/00007435-200212000-00004 12466716

[ref12] Ellen J. M. Boyer C. B. Tschann J. M. Shafer M.-a. (1996). Adolescents' perceived risk for STDs and HIV infection. J. Adolesc. Health 18, 177–181. doi: 10.1016/1054-139X(94)00103-L 8777193

[ref13] Ferguson Y. O. Quinn S. C. Eng E. Sandelowski M. (2006). The gender ratio imbalance and its relationship to risk of HIV/AIDS among African American women at historically black colleges and universities. AIDS Care 18, 323–331. doi: 10.1080/09540120500162122, PMID: 16809109

[ref14] Flash C. A. Adegboyega O. O. Yu X. Avalos C. Johnson S. Mayer K. H.. (2018). Correlates of linkage to HIV Preexposure prophylaxis among HIV-testing clients. J. Acquir. Immune Defic. Syndr. 77, 365–372. doi: 10.1097/QAI.0000000000001605 29474256 PMC5949071

[ref15] Ford C. L. Daniel M. Miller W. C. (2006). High rates of HIV testing despite low perceived HIV risk among African-American sexually transmitted disease patients. J. Natl. Med. Assoc. 98, 841–844, PMID: 16775904 PMC2569408

[ref16] Gerend M. A. Magloire Z. F. (2008). Awareness, knowledge, and beliefs about human papillomavirus in a racially diverse sample of young adults. J. Adolesc. Health 42, 237–242. doi: 10.1016/j.jadohealth.2007.08.022 18295131

[ref17] Gilbert L. Goddard-Eckrich D. Chang M. Hunt T. Wu E. Johnson K.. (2021). Effectiveness of a culturally tailored HIV and sexually transmitted infection prevention intervention for black women in community supervision programs: a randomized clinical trial. JAMA Netw. Open 4:e215226. doi: 10.1001/jamanetworkopen.2021.5226, PMID: 33835175 PMC8035652

[ref18] Grimley D. M. Hook E. W. 3rd. (2009). A 15-minute interactive, computerized condom use intervention with biological endpoints. Sex. Transm. Dis. 36, 73–78. doi: 10.1097/OLQ.0b013e31818eea81, PMID: 19125141

[ref19] Henry W. J. (2008). Black female millennial college students: dating dilemmas and identity development. Multicult. Educ. 16, 17–21.

[ref20] Henry W. J. (2013). The black gender gap: a commentary on intimacy and identity issues of black college women. Professional Counselor 3, 185–193. doi: 10.15241/wjh.3.3.185

[ref21] Hill M. J. Heads A. M. Green C. Suchting R. Stotts A. L. (2022). Pilot testing the effectiveness of whether a survey-driven tablet-based intervention increased willingness of black women to attend to an initial PrEP clinic visit: the protocol for the pilot randomized controlled trial design and methods. Contemp Clin Trials Commun. 29:100985. doi: 10.1016/j.conctc.2022.100985, PMID: 36092974 PMC9450123

[ref22] Hill M. J. Heads A. M. Suchting R. Stotts A. L. (2023). A survey with interventional components delivered on tablet devices versus usual care to increase pre-exposure prophylaxis uptake among cisgender black women: a pilot randomized controlled trial. BMC Infect. Dis. 23:57. doi: 10.1186/s12879-023-08019-z, PMID: 36707778 PMC9881522

[ref23] Ho G. Y. Bierman R. Beardsley L. Chang C. J. Burk R. D. (1998). Natural history of cervicovaginal papillomavirus infection in young women. N. Engl. J. Med. 338, 423–428. doi: 10.1056/NEJM199802123380703, PMID: 9459645

[ref24] Kalichman S. C. Cain D. (2005). Perceptions of local HIV/AIDS prevalence and risks for HIV/AIDS and other sexually transmitted infections: preliminary study of intuitive epidemiology. Ann. Behav. Med. 29, 100–105. doi: 10.1207/s15324796abm2902_4, PMID: 15823783

[ref25] Khan M. R. Kaufman J. S. Pence B. W. Gaynes B. N. Adimora A. A. Weir S. S.. (2009). Depression, sexually transmitted infection, and sexual risk behavior among young adults in the United States. Arch. Pediatr. Adolesc. Med. 163, 644–652. doi: 10.1001/archpediatrics.2009.95, PMID: 19581548 PMC2796823

[ref26] Kiviniemi M. T. Orom H. Waters E. A. McKillip M. Hay J. L. (2018). Education-based disparities in knowledge of novel health risks: the case of knowledge gaps in HIV risk perceptions. Br. J. Health Psychol. 23, 420–435. doi: 10.1111/bjhp.12297, PMID: 29388364 PMC5882541

[ref27] Mays V. M. Cochran S. D. (1988). Issues in the perception of AIDS risk and risk reduction activities by Black and Hispanic/Latina women. Am. Psychol. 43, 949–957. doi: 10.1037/0003-066X.43.11.949 3214007 PMC4196373

[ref28] Nydegger L. A. Dickson-Gomez J. Ko Ko T. (2020). A longitudinal, qualitative exploration of perceived HIV risk, healthcare experiences, and social support as facilitators and barriers to PrEP adoption among black women. AIDS Behav. 25, 582–591. doi: 10.1007/s10461-020-03015-9 PMC7855297 32886220

[ref29] Nydegger L. A. Dickson-Gomez J. Ko T. K. (2020). Structural and syndemic barriers to PrEP adoption among black women at high risk for HIV: a qualitative exploration. Cult. Health Sex. 23, 659–673. doi: 10.1080/13691058.2020.1720297 32212993 PMC7529643

[ref30] Patel A. S. Goparaju L. Sales J. M. Mehta C. C. Blackstock O. J. Seidman D.. (2019). Brief report: PrEP eligibility among at-risk women in the southern United States: associated factors, awareness, and acceptability. J. Acquir. Immune Defic. Syndr. 80, 527–532. doi: 10.1097/QAI.0000000000001950 30649036 PMC6519058

[ref31] Pharr J. Enejoh V. Mavegam B. O. Olutola A. Karick H. Ezeanolue E. E. (2015). Relationship between health locus of control and risky sexual behaviors among Nigerian adolescents. J AIDS. Clin. Res. 28, 672–676. doi: 10.1080/09540121.2015.1120853 PMC4712957 26779383

[ref32] Prata N. Morris L. Mazive E. Vahidnia F. Stehr M. (2006). Relationship between HIV risk perception and condom use: evidence from a population-based survey in Mozambique. Int. Fam. Plan. Perspect. 32, 192–200. doi: 10.1363/3219206, PMID: 17237016

[ref33] Pratt M. C. Jeffcoat S. Hill S. V. Gill E. Elopre L. Simpson T.. (2022). We feel like Everybody's going to judge us": Black adolescent girls’ and young women’s perspectives on barriers to and opportunities for improving sexual health care, including PrEP, in the southern U.S. J. Int. Assoc. Provid. AIDS Care 21:7327. doi: 10.1177/23259582221107327 PMC9201301 35699978

[ref34] Pyra M. Johnson A. K. Devlin S. Uvin A. Z. Irby S. Stewart E.. (2022). HIV pre-exposure prophylaxis use and persistence among black ciswomen: "women need to protect themselves, period". J. Racial Ethn. Health Disparities 9, 820–829. doi: 10.1007/s40615-021-01020-9, PMID: 33733424 PMC7968858

[ref35] Rodriguez DK, H. How Race, Ethnicity, and gender impact your Life’s worth: Discrimination in civil damage awards. Silicon Valley, CA: Economic Justice Project. (2018).

[ref36] Rosenthal L. Lobel M. (2016). Stereotypes of black American women related to sexuality and motherhood. Psychol. Women Q. 40, 414–427. doi: 10.1177/0361684315627459, PMID: 27821904 PMC5096656

[ref37] Salisu M. A. Dacus J. D. (2021). Living in a paradox: how older single and widowed black women understand their sexuality. J. Gerontol. Soc. Work. 64, 303–333. doi: 10.1080/01634372.2020.1870603, PMID: 33402054 PMC8026708

[ref38] Sewell W. C. Blankenship S. A. (2019). Perceived HIV risk as a predictor of sexual risk behaviors and discrimination among high-risk women. AIDS Care 31, 675–680. doi: 10.1080/09540121.2018.1533234, PMID: 30318900 PMC6443477

[ref39] Sly D. F. Quadagno D. Harrison D. F. Eberstein I. Riehman K. (1997). The association between substance use, condom use and sexual risk among low-income women. Fam. Plan. Perspect. 29, 132–136. doi: 10.2307/2953336 9179583

[ref40] Smith E. P. Witherspoon D. P. Bhargava S. Bermudez J. M. (2019). Cultural values and behavior among African American and European American children. J. Child Fam. Stud. 28, 1236–1249. doi: 10.1007/s10826-019-01367-y, PMID: 31871395 PMC6927402

[ref41] Thapa R. van Teijlingen E. Regmi P. R. Heaslip V. (2021). Caste exclusion and health discrimination in South Asia: a systematic review. Asia Pac. J. Public Health 33, 828–838. doi: 10.1177/10105395211014648, PMID: 34024157 PMC8592103

[ref42] Tillerson K. (2008). Explaining racial disparities in HIV/AIDS incidence among women in the U.S.: a systematic review. Stat. Med. 27, 4132–4143. doi: 10.1002/sim.3224, PMID: 18551508 PMC2684462

[ref43] Wagstaff D. A. Kelly J. A. Perry M. J. Sikkema K. J. Solomon L. J. Heckman T. G.. (1995). Multiple partners, risky partners and HIV risk among low-income urban women. Fam. Plan. Perspect. 27, 241–245. doi: 10.2307/2136176, PMID: 8666088

[ref44] Waterman A. S. (1981). Individualism and interdependence. Am. Psychol. 36, 762–773. doi: 10.1037/0003-066X.36.7.762

[ref45] Willie T. C. Monger M. Nunn A. Kershaw T. Stockman J. K. Mayer K. H.. (2021). "PrEP's just to secure you like insurance": a qualitative study on HIV pre-exposure prophylaxis (PrEP) adherence and retention among black cisgender women in Mississippi. BMC Infect. Dis. 21:1102. doi: 10.1186/s12879-021-06786-1, PMID: 34702165 PMC8549215

[ref46] Younge S. N. Salem D. Bybee D. (2010). Risk revisited: the perception of HIV risk in a community sample of low-income African American women. J. Black Psychol. 36, 49–74. doi: 10.1177/0095798408320633

